# The Effects of Semaglutide on Inflammation and Immune Activation in HIV-associated Lipohypertrophy

**DOI:** 10.1093/ofid/ofaf152

**Published:** 2025-03-20

**Authors:** Nicholas T Funderburg, Allison Ross Eckard, Qian Wu, Abdus Sattar, Kate Ailstock, Morgan Cummings, Danielle Labbato, Grace A McComsey

**Affiliations:** Division of Medical Laboratory Sciences, School of Health and Rehabilitation Sciences, The Ohio State University, Columbus, Ohio, USA; Departments of Pediatrics and Medicine, Medical University of South Carolina, Charleston, South Carolina, USA; School of Medicine, Case Western Reserve University, Cleveland, Ohio, USA; School of Medicine, Case Western Reserve University, Cleveland, Ohio, USA; Division of Medical Laboratory Sciences, School of Health and Rehabilitation Sciences, The Ohio State University, Columbus, Ohio, USA; Division of Medical Laboratory Sciences, School of Health and Rehabilitation Sciences, The Ohio State University, Columbus, Ohio, USA; Departments of Pediatrics and Medicine, University Hospitals of Cleveland, Cleveland, Ohio, USA; School of Medicine, Case Western Reserve University, Cleveland, Ohio, USA; Departments of Pediatrics and Medicine, University Hospitals of Cleveland, Cleveland, Ohio, USA

**Keywords:** C-reactive protein, HIV-1, inflammation, interleukin-6, semaglutide

## Abstract

**Background:**

Cardiovascular and metabolic comorbidities are common in people with HIV (PWH) and are linked to chronic inflammation and immune activation. We assessed the effects of semaglutide on plasma markers of immune activation/inflammation that are known to be increased in PWH and are associated with morbidity and mortality in this population.

**Methods:**

We conducted a single-site, randomized, double-blinded, placebo-controlled trial of virologically suppressed, nondiabetic PWH ≥18 years of age on stable antiretroviral therapy with body mass index ≥ 25 kg/m^2^, increased waist circumference/waist-to-hip ratio, and subjective increased abdominal girth after antiretroviral therapy initiation (clinicaltrials.gov: NCT04019197). Participants were randomized 1:1 to 32 weeks of semaglutide (8-week titration + 24 weeks of 1.0 mg weekly subcutaneous injection) or matching placebo. Signed-rank tests were used to determine changes over 32 weeks in soluble markers and cellular phenotypes of inflammation/immune activation within groups; semaglutide effects were assessed using linear or quantile regression analyses.

**Results:**

A total of 108 participants were enrolled and evenly randomized to semaglutide versus placebo. Eight (15%) in each group withdrew prematurely. Thirty-two weeks of semaglutide treatment reduced baseline levels of C-reactive protein, interleukin-6, and soluble CD163 (all *P* < .02) and trended to reduce levels of sCD14 (*P* = .08). Circulating monocyte proportions and T-cell phenotypes were not altered by semaglutide.

**Conclusions:**

In this randomized controlled trial of semaglutide in PWH, we report significant decreases in markers of inflammation that are associated with morbidity and mortality in this population. These results add to the growing literature demonstrating the anti-inflammatory effects of semaglutide. Further studies in PWH are warranted.

## Teaser

People with HIV are at increased risk for cardiometabolic complications. Here, we demonstrate that treatment with the glucagon-like peptide 1 receptor agonist semaglutide reduced levels of pro-inflammatory biomarkers that have been linked to morbidity and mortality in people with HIV.

People with HIV (PWH) often experience cardiometabolic complications, including lipohypertrophy, dyslipidemias, increased risk of diabetes, and coronary artery disease [1, 2]. These comorbidities have been linked to chronic inflammation and immune activation. As people age with HIV, changes in metabolic indices and persistent inflammation likely contribute to an increased risk of age-related comorbidities, including cardiovascular disease (CVD) [3]. Furthermore, PWH also tend to have higher rates of dyslipidemia, insulin resistance, alterations in gut hormone secretion, and decreases in muscle mass and function compared to people without HIV [4–6].

Multiple plasma biomarkers of immune activation and inflammation (eg, interleukin-6 [IL-6], high-sensitivity C-reactive protein [hsCRP], soluble CD14 [sCD14]) have been linked to morbidity and mortality in PWH, including events related to cardiometabolic disease [7–10]. Several clinical studies have sought to reduce CVD risk and lower levels of inflammation in PWH, but these studies have had mixed results [11]. Recently, the randomized trial to prevent vascular events in HIV (REPRIEVE), in which PWH received the lipid-lowering agent, pitavastatin, demonstrated that statin treatment reduced biomarkers of vascular inflammation [12] and CVD events in this population [13]. These results advanced our previous findings in Stopping Atherosclerosis and Treating Unhealthy bone with RosuvastatiN in HIV (SATURN-HIV) in which PWH receiving rosuvastatin, compared to placebo, had significant decreases in monocyte activation and inflammation [14, 15]. Although these studies provide promising results, further trials aimed at reducing cardiometabolic risk in PWH are needed because the REPREIVE study did not completely reduce the elevated risk of CVD events in PWH.

Glucagon-like peptide 1 receptor agonists reduce weight and improve insulin sensitivity in the general population [16–18]. These molecules, including semaglutide, stimulate the glucose-dependent release of insulin from pancreatic islets, which inhibits glucagon release and slows gastric emptying. Recently, in a placebo-controlled trial, we reported that 32 weeks of semaglutide resulted in significant decreases in abdominal visceral (β −30.82 cm², 95% confidence interval [CI], −50.13 to −11.51; % change −30.6%) and subcutaneous (β −42.01 cm², 95% CI, −75.49 to −8.52; % change −11.2%) adipose tissue (AT) and in total body fat (natural logarithmic −0.21 kg, 95% CI, −0.33 to −0.08; % change −18.9%) among PWH with HIV-associated lipohypertrophy [19]. We also reported improvements in measures of glucose metabolism and insulin resistance (ie, HbA_1C_, insulin, and homeostatic model of insulin resistance) and decreases in very-low-density lipoprotein cholesterol and triglycerides and an increase in high-density lipoprotein cholesterol in participants receiving semaglutide [19]. Here, we present the preplanned outcomes of 32 weeks of semaglutide treatment on plasma markers of inflammation and on immune cell populations in PWH with HIV-associated lipohypertrophy.

## MATERIAL AND METHODS

### Study Design

This is a randomized, double-blind, placebo-controlled phase IIb clinical trial approved by the institutional review board of University Hospitals of Cleveland (IORG000040; clinicaltrials.gov: NCT04019197). As previously described [19], participants with HIV-associated lipohypertrophy were enrolled at a single site (University Hospitals Cleveland Medical Center, Cleveland, Ohio, USA) and were randomized to receive 32 weeks of once-weekly subcutaneous (SC) semaglutide or matching placebo. Study statistician (A.S.) provided randomization allocation sequences generated from an online software program (sealedenvelope.com) with a 1:1 ratio and block size of 6; key personnel, study staff, participants, and participants’ medical providers were masked to treatment assignment. Participants received study drug free of charge.

### Participants

Inclusion and exclusion criteria have been described previously [19], but, in brief, eligible participants were ≥18 years old with documented HIV-1 infection, cumulative antiretroviral therapy (ART) duration ≥1 year, receiving a stable ART regimen for ≥12 weeks, and HIV-1 RNA <400 copies/mL for ≥6 months before entry. Participants required subjective observation of increased abdominal girth occurring after ART initiation, and designated cutoffs for waist circumference and waist-to-hip ratio of >95 cm and >0.94 cm, respectively, for men, and >94 cm and >0.88 cm, respectively, for women. Participants had to have a body mass index ≥25 kg/m^2^. Known history of diabetes or CVD was exclusionary.

### Procedures

Qualifying participants entered an initial 4-week lead-in screening phase consisting of weekly study visits (weeks −4, −3, −2, −1). At each visit, participants underwent targeted physical examinations, completed detailed dietary and physical activity questionnaires, and received standardized dietary and physical activity advice from a registered clinical dietician. This phase was designed to assess stability of lifestyle habits, anthropometric measurements, and ability to complete study requirements and adhere to weekly study visits.

As previously detailed [19], participants who successfully completed screening were enrolled into the interventional phase and randomized to receive semaglutide or matching placebo (week 0/entry). During the titration phase, participants were given weekly 0.25 mg SC semaglutide injections for 4 weeks, followed by weekly 0.5 mg SC semaglutide injections (or matching placebo) for another 4 weeks. After the titration phase, participants received 1.0 mg SC semaglutide weekly for 24 weeks or matching placebo. At the time of study initiation, June 2019, this was the Food and Drug Administration–approved dose for semaglutide.

### Plasma Biomarker Measurements

Plasma was isolated from blood samples collected in EDTA anticoagulant tubes and were stored at −80 °C and batched until processing without a prior thaw. Then, using an enzyme-linked immunosorbent assay, we measured the following plasma biomarkers: sCD14, soluble CD163 (sCD163), tumor necrosis factor receptors I and II, vascular and intercellular cell adhesion molecule 1 (VCAM-1 and ICAM-1), hsCRP, and IL-6, all from R & D Systems, Minneapolis, Minnesota. Oxidized low-density lipoprotein (oxidized LDL) and D-dimer levels were also measured by enzyme-linked immunosorbent assay (Mercodia, Uppsala, Sweden, and Diagnostic Stago Inc., Parsippany, NJ, respectively). The intra-assay variability ranged between 4% and 8% and inter-assay variability was less than 10% for all markers. All assays were performed in Dr. Funderburg's laboratory at Ohio State University, Columbus, Ohio, and laboratory personnel were blinded to group assignments.

### Flow Cytometry

Cellular markers of immune activation were measured on cryopreserved peripheral blood mononuclear cells by flow cytometry using a Miltenyi MACSQuant flow cytometer (Miltenyi Biotec, Bergisch Gladbach, Germany). Monocyte subsets were identified based on CD14 and CD16 expression, including CD14^+^ CD16^+^ (inflammatory) and CD14^dim^ CD16^+^ (patrolling) monocytes, and were quantified as a percentage of the overall monocyte population. Activated CD4^+^ and CD8^+^ lymphocytes expressing both CD38 and human leukocyte antigen (HLA)-DR were quantified as a percentage of the overall CD4^+^ and CD8^+^ lymphocyte population, respectively. Senescence/exhaustion was measured among T cells based on expression of CD28 and CD57 (CD28^–^CD57^+^) and on positive expression of PD-1.

### Statistical Analyses

After rigorous data quality checks using descriptive analyses and graphs, we plotted biomarkers for visualization over the study period. Many of the inflammatory biomarkers differed between the 2 groups (semaglutide vs placebo) at baseline. Depending on the distribution of the data, we used either linear regression or quantile (median) regression models to estimate the effect of semaglutide on the outcome variables or biomarkers. Because of the differences in biomarkers at baseline, we controlled for heterogeneities by adding baseline values of the outcome variables as covariates. We also controlled for known confounding covariates such as age, sex, and smoking status in the regression analyses. All analyses were performed using intention-to-treat principles based on randomized treatment assignment, including all available data. Between-group comparisons were conducted using either 2-sample *t*-tests or Wilcoxon rank-sum tests, as appropriate. We used statistical software Stata 18.0 for all quantitative analyses and R 4.3.3 for graphing.

## RESULTS

Potential participants were screened for eligibility (N = 154), and 108 participants were enrolled and randomized to receive semaglutide or placebo. Eight participants in each group withdrew from the study before the 32-week endpoint. Participant demographic and clinical information have been described in detail previously [19] and are in Table 1. All but 1 participant had ART-suppressed HIV-1 viremia (HIV RNA <400 copies/mL) at baseline, and all participants achieved and maintained viral suppression during the trial. Representation of ART regimens is listed in Table 1. Study arms were generally similar on demographic and clinical indices, but the semaglutide arm had a greater proportion of men (70% to 50%, Table 1) and the placebo group had a greater proportion of current smokers (43% to 28%, Table 1). There were no differences in statin use by participants between arms and treatment had no significant effect on dietary changes (as measured by energy intake, *P* > .80, not shown). We have previously reported that semaglutide reduced total body fat, trunk/abdominal fat, limb fat, and weight in this study population [19].

**Table 1.** Demographic and Clinical Information on Study Participants

	Semaglutide (n = 54)	Placebo (n = 54)
Demographics		
Age, y	53 (40, 57)	53 (41, 57)
On statins		
No	52 (96.30%)	50 (92.59%)
Yes	2 (3.70%)	4 (7.41%)
Smoking		
Never/past	39 (72.22%)	31 (57.41%)
Current	15 (27.78%)	23 (42.59%)
Sex		
Male	38 (70.0%)	27 (50.0%)
Female	16 (30%)	27 (50.0%)
Race		
Black	33 (61.1%)	34 (60.0%)
White	20 (37.0%)	18 (33.3%)
Biracial	0 (0.0%)	2 (3.7%)
Native American	1 (1.9%)	0 (0.0%)
Hispanic ethnicity	4 (7.0%)	5 (9.0%)
HIV variables		
CD4^+^ T-cell count, cells/µL	826 (407, 1058)	793 (579, 994)
HIV duration, mo	203 (118, 305)	226 (155, 282)
Antiretroviral therapy duration, mo	168 (98, 230)	148 (98, 198)
Current INSTI use	45 (83.0%)	43 (80.0%)
Current protease inhibitor use	10 (19.0%)	8 (15.0%)
Current tenofovir alafenamide use	43 (80.0%)	37 (69.0%)

Abbreviation: INSTI, integrase strand transfer inhibitors.

**Figure 1.**
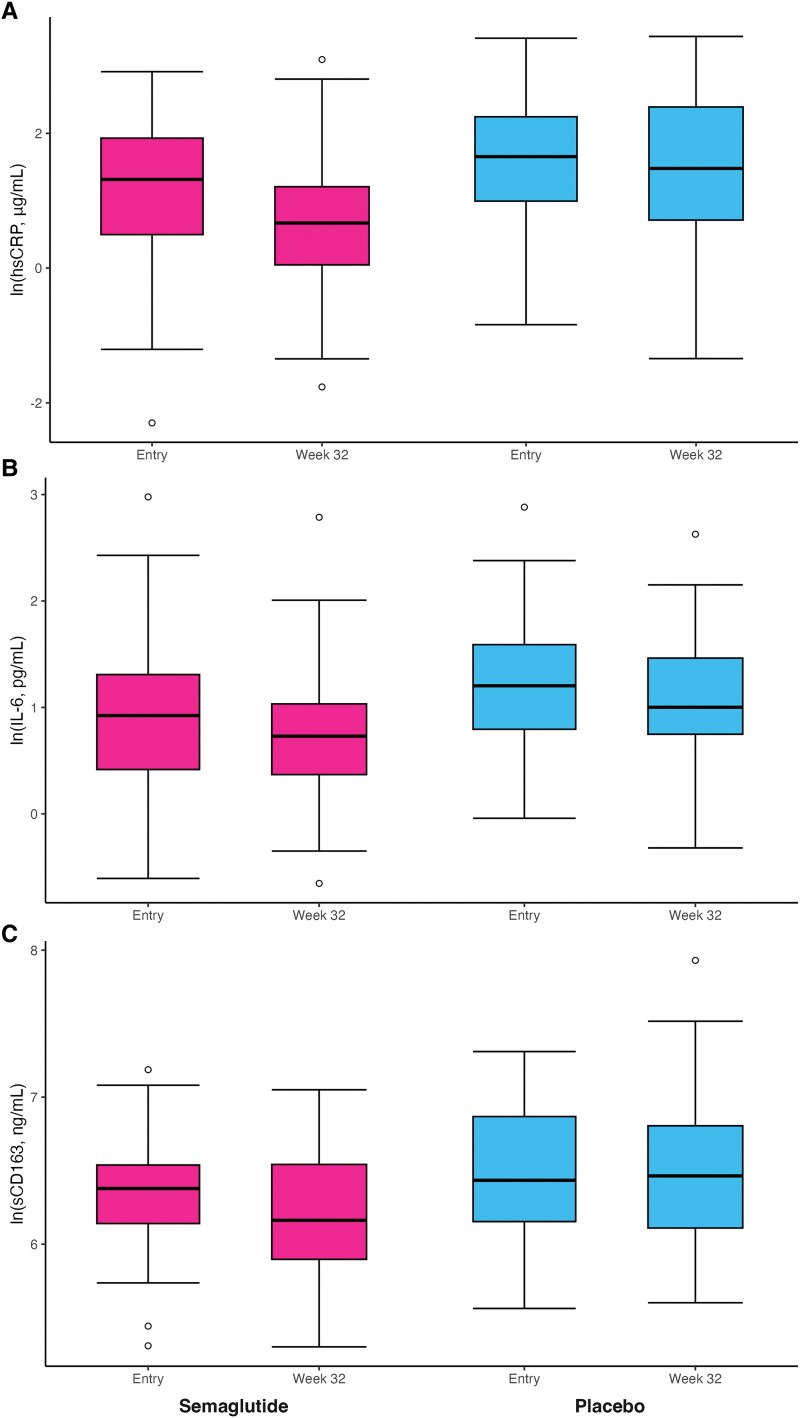
Semaglutide treatment reduces levels of inflammation and monocyte activation in PWH. Levels of (A) high-sensitivity C-reactive protein (hsCRP), (B) interleukin-6 (IL-6), and (C) soluble CD163 (sCD163) were measured by enzyme-linked immunosorbent assay (ELISA) in participants receiving semaglutide (N = 54) or placebo (N = 54) at baseline and week 32. The box plot shows the 5-number summary of a set of data. The hollow circles in the plot represent outliers that fall outside 1.5 times the interquartile range (IQR) from the quartiles. The whiskers extend to the smallest and largest values within 1.5 times the IQR from the first and third quartiles, respectively. The bottom of the box shows the first quartile (the value at 25% of the data). There is a black line in the middle of the box, which is the median of the data. The top of the box displays the third quartile (the value at 75% of the data).

Profiles of circulating immune cells and levels of plasma markers of immune cell and endothelial cell activation, inflammation, and coagulation were generally similar between arms at baseline (Supplementary Table 1 and Table 2), but levels of hsCRP, IL-6, and sICAM-1 were all higher in the placebo group compared to levels in the semaglutide group. Proportions of monocyte subsets (CD14^+^CD16^−^, CD14^+^CD16^+^, and CD14^Dim^CD16^+^) and proportions of activated (CD38^+^HLA-DR^+^) and senescent/exhausted (CD57^+^CD28^−^ or PD-1^+^) CD4^+^ and CD8^+^ T cells did not change in either arm over 32 weeks (Supplementary Table 2). Treatment with semaglutide resulted in significant decreases in the absolute levels of hsCRP (−1.17 µg/mL, *P* = .008, −47.73%, *P* = .05), IL-6 (−0.39 pg/mL, *P* = .016, −17.73%, *P* = .15) and sCD163 (−61.3 ng/mL, *P* = .005, −9.54%, *P* = .014. Figure 1*A*-*C*). Absolute levels of sCD14 (−130.83 ng/mL, *P* = .08, −8.63%, *P* = .27) and oxidized LDL (−4.29 U/mL, *P* = .126, −9.07%, *P* = .28) tended to decrease with semaglutide treatment but these changes did not reach statistical significance (Table 3). After performing a Benjamini-Hochberg correction for multiple comparisons, changes in hsCRP, IL-6, and sCD163 remained significant within the semaglutide arm. Both the absolute change and percentage change of hsCRP were significantly different following 32 weeks of treatment with semaglutide compared to treatment with placebo. We did not detect a significant change in CD4^+^ T cells in either study arm. There were no significant changes in other biomarkers within the semaglutide arm, and no biomarkers changed significantly in the placebo arm. After stratification according to the baseline median hsCRP, there were significant changes in IL-6 and sCD163 in the above-median group from baseline, and this change in IL-6 was statistically significantly greater than the change in IL-6 in the below-median in the treatment group (Table 4). Following correction for multiple comparisons, changes in IL-6 and sCD163 remained significant in the semaglutide group with high baseline hsCRP levels.

**Table 2.** Baseline Characteristics of Inflammatory Markers in 2 Groups

	Semaglutide (n = 54)	Placebo (n = 54)	*P* ^a^	BH
hsCRP, µg/mL	3.73 (1.60–7.04)	5.23 (2.66–9.46)	.01^b^	0.02
IL-6, pg/mL	2.52 (1.46–3.76)	3.33 (2.16–5.02)	.02^b^	0.04
sICAM-1, ng/mL	234.92 (194.11–262.55)	253.04 (205.00–335.65)	.04^b^	0.06
sCD14, ng/mL	1618.15 (1404.96–2024.07)	1895.51 (1577.47–2129.98)	.13	0.08
sCD163, ng/mL	588.90 (461.40–695.61)	622.95 (469.10–961.60)	.20	0.10
sVCAM-1, ng/mL	736.79 (643.68–880.07)	681.31 (619.00–824.68)	.29	0.12
sTNFR-I, ng/mL	0.86 (0.70–1.16)	0.99 (0.74–1.16)	.34	0.14
D-dimer, µg/mL	0.31 (0.19–0.51)	0.38 (0.27–0.54)	.49	0.16
sTNFR-II, ng/mL	2.26 (2.01–2.89)	2.41 (1.99–2.86)	.57	0.18
oxLDL, U/L	57.79 (45.15–78.87)	58.73 (46.49–81.31)	.92	0.20

*P* values were obtained from either 2-sample *t*-tests or Wilcoxon rank-sum tests.

Abbreviations: BH, Benjamini-Hochberg; hsCRP, high-sensitivity C-reactive protein; IL-6, interleukin 6; oxLDL, oxidized low-density lipoprotein; sICAM, soluble intercellular cell adhesion molecule 1; sTNFR, soluble tumor necrosis factor receptor; sVCAM, soluble vascular cell adhesion molecule 1.

^a^ *t*-test or rank-sum test was applied to continuous variables depending on the distribution.

^b^Significant *P* values after BH false discovery rate 0.20 correction.

**Table 3.** Absolute and Percent Changes in the Inflammatory Markers Over the 32-week Study Period

	Semaglutide		Placebo			
	Median (IQR)	*P* ^b^	Median (IQR)	*P* ^b^	*P* ^a^	BH
A. Absolute changes over 32 wk^c^						
hsCRP, µg/mL	−1.17 (−3.09–0.15)	.01^d^	−0.11 (−1.30 to 2.37)	.81	.02	0.02
sCD163, ng/mL	−61.30 (−162.44–35.78)	.00^d^	−15.23 (−107.35 to 79.16)	.75	.06	0.04
IL-6, pg/mL	−0.39 (−1.62–0.30)	.02^d^	−0.25 (−1.66 to 0.74)	.25	.43	0.06
sTNFR-II, ng/mL	−0.16 (−0.70–0.35)	.39	0.07 (−0.68 to 0.70)	.81	.47	0.08
D-dimer, µg/mL	0.05 (−0.14–0.19)	.45	0.02 (−0.12 to 0.14)	.95	.53	0.10
sCD14, ng/mL	−130.83 (−446.01–136.27)	.09	−106.11 (−364.40 to 296.23)	.36	.62	0.12
sVCAM-1, ng/mL	2.44 (−94.83–81.83)	.78	−21.21 (−68.58 to 93.50)	.74	.65	0.14
oxLDL, U/L	−4.29 (−21.38 to 7.42)	.13	2.16 (−20.14 to 14.75)	.74	.82	0.16
sICAM-1, ng/mL	−4.99 (−36.54 to 33.00)	.85	−6.85 (−36.64 to 31.19)	.63	.82	0.18
sTNFR-I, ng/mL	−0.07 (−0.23 to 0.19)	.58	−0.06 (−0.24 to 0.16)	.63	.96	0.20
B. % Changes over 32 wk^c^						
hsCRP, µg/mL	−47.73 (−66.77 to 10.88)	.05	−8.74 (−46.29 to 35.86)	.69	.04	0.02
sCD163, ng/mL	−9.54 (−26.14 to 4.06)	.01^d^	−2.47 (−18.85 to 10.81)	.89	.07	0.04
sTNFR-II, ng/mL	−8.30 (−28.19 to 19.49)	.69	2.52 (−25.87 to 37.77)	.33	.31	0.06
oxLDL, U/L	−9.07 (−28.03 to 14.60)	.28	3.39 (−26.19 to 29.21)	.73	.37	0.08
IL-6, pg/mL	−17.73 (−39.94 to 20.03)	.15	−11.55 (−40.86 to 25.02)	.41	.56	0.10
sVCAM-1, ng/mL	0.38 (−11.43 to 11.71)	.68	−3.48 (−10.68 to 11.69)	.80	.61	0.12
sCD14, ng/mL	−8.63 (−21.60 to 8.10)	.27	−5.48 (−19.40 to 16.16)	.93	.64	0.14
D-dimer, µg/mL	14.96 (−32.77 to 86.40)	.05	7.17 (−29.81 to 59.15)	.20	.65	0.16
sICAM-1, ng/mL	−2.93 (−18.89 to 14.33)	.86	−3.41 (−14.61 to 16.10)	.76	.74	0.18
sTNFR-I, ng/mL	−7.50 (−20.56 to 28.45)	.88	−6.08 (−19.62 to 24.70)	.98	.92	0.20

Abbreviations: BH, Benjamini-Hochberg; hsCRP, high-sensitivity C-reactive protein; IL-6, interleukin 6; oxLDL, oxidized low-density lipoprotein; sICAM, soluble intercellular cell adhesion molecule 1; sTNFR, soluble tumor necrosis factor receptor; sVCAM, soluble vascular cell adhesion molecule 1.

^a^Between group *P* values.

^b^Within group *P* values.

^c^The absolute changes were computed as (week 32 – baseline) and the percentage changes were computed as ((week 32 – baseline)/baseline)×100.

^d^Significant *P* values after BH false discovery rate 0.20 correction.

**Table 4.** Effects of Semaglutide on the Inflammation Markers for Those Above vs Below the Median hsCRP (4.04 µg/mL) at Baseline

	Above Median hsCRP Group (*P*)		Below Median hsCRP Group (*P*)		Between Group *P*	BH
	N = 24		N = 30			
IL-6, pg/mL	−1.27 (−2.35 to 0.14)	.01^a^	0.06 (−0.58 to 0.49)	.95	.01^a^	0.02
sCD163, ng/mL	−96.66 (−152.55 to 11.05)	.00^a^	−24.15 (−162.44 to 44.14)	.34	.20	0.04
oxLDL, U/L	−7.65 (−21.38 to 7.42)	.14	−2.71 (−17.51 to 6.97)	.68	.36	0.07
D-dimer, µg/mL	0.00 (−0.15 to 0.09)	.99	0.05 (−0.10 to 0.23)	.31	.43	0.09
sCD14, ng/mL	−158.82 (−522.90 to 309.25)	.11	−30.65 (−199.45 to 98.73)	.37	.43	0.11
sTNFR-I, ng/mL	−0.09 (−0.21 to 0.16)	.32	−0.05 (−0.23 to 0.19)	.97	.55	0.13
sVCAM-1, ng/mL	−10.12 (−94.83 to 70.79)	.79	15.17 (−86.01 to 81.83)	.68	.60	0.16
sICAM-1, ng/mL	−11.82 (−39.45 to 33.00)	.66	−4.47 (−30.24 to 32.20)	.95	.70	0.18
sTNFR-II, ng/mL	−0.20 (−0.54 to 0.22)	.29	0.05 (−0.71 to 0.36)	.66	.80	0.20

Abbreviations: BH, Benjamini-Hochberg; hsCRP, high-sensitivity C-reactive protein; IL-6, interleukin 6; oxLDL, oxidized low-density lipoprotein; sICAM, soluble intercellular cell adhesion molecule 1; sTNFR, soluble tumor necrosis factor receptor; sVCAM, soluble vascular cell adhesion molecule 1.

^a^Significant *P* values after BH false discovery rate 0.20 correction.

Treatment effects of semaglutide were significant in regression analyses (β coefficient [95% CI]) for log hsCRP (−0.51 [−0.87, −0.15]; *P* = .006) with trends in log IL-6 (−0.21 [−0.44, 0.02]; *P* = .074) and log sCD14 (−0.07 [−0.19, 0.04]; *P* = .186). Because of differences in the study arms at baseline (ie, age, sex, smoking status), we adjusted for these values, and following adjustment, the effect of semaglutide treatment on hsCRP remained significant (Table 5). There were no significant correlations between changes in hsCRP and changes in weight or changes in abdominal visceral adipose tissue (VAT) (Supplementary Tables 3A and 3B), however, we did find that changes in VAT were associated with changes in IL-6 (β = –0.02; 95% CI, −0.03 to −0.004; *P* = .012), but this association was insignificant in the semaglutide group in a subgroup analysis. We also performed mediation analyses by focusing specifically on the natural indirect effects of changes in inflammatory markers and changes in glucose, HbA1c, and insulin and none of the natural indirect effects were statistically significant, suggesting that changes in these markers were not associated with changes in inflammatory markers in this study.

**Table 5.** Effects of Semaglutide on the Inflammation Markers by Linear/Median Regression Adjusting for Baseline Outcome Values, Age, Sex, and Smoking

	Coef. (β)	SE (β)	*P V*alue	95% CI	BH	% change
hsCRP, µg/mL	−0.51	0.18	.01^a^	−.87 to −.15	0.02	−39.92
sCD163, ng/mL	−0.13	0.07	.05	−0.26 to .001	0.04	−12.10
IL-6, pg/mL	−0.21	0.12	.07	−.44 to .02	0.06	−18.84
sCD14, ng/mL	−0.07	0.06	.19	−.19 to .04	0.08	−7.22
oxLDL, U/L	−0.11	0.08	.19	−.27 to .06	0.10	−10.30
sTNFR-II, ng/mL	−0.09	0.07	.20	−.23 to .05	0.12	−8.71
D-dimer, µg/mL	0.10	0.12	.41	−.14 to .33	0.14	10.36
sVCAM-1, ng/mL	0.01	0.04	.75	−.07 to .09	0.16	1.28
sTNFR-I, ng/mL	−0.01	0.06	.91	−.13 to .12	0.18	−0.70
sICAM-1, ng/mL	−0.001	0.05	.98	−.10 to .09	0.20	−0.14

Abbreviations: BH, Benjamini-Hochberg; hsCRP, high-sensitivity C-reactive protein; IL-6, interleukin 6; oxLDL, oxidized low-density lipoprotein; sICAM, soluble intercellular cell adhesion molecule 1; sTNFR, soluble tumor necrosis factor receptor; sVCAM, soluble vascular cell adhesion molecule 1.

^a^No significant *P* values after BH false discovery rate 0.20 correction.

## DISCUSSION

People with HIV are at increased risk of cardiometabolic diseases (eg, obesity, atherosclerosis, type 2 diabetes [T2D]) compared to people without HIV (PWoH) and several studies are aimed at reducing this risk. We have reported previously that semaglutide reduced waist circumference and abdominal visceral and subcutaneous fat in this study population [19]. An open-label trial of semaglutide in PWH reported improvement in intrahepatic triglycerides and a reduction in metabolic dysfunction-associated steatotic liver disease in study participants [20]. A retrospective study also demonstrated that GLP-1RAs reduced weight, body mass index and HbA1c in PWH [21]. Alterations in metabolic profiles and persistent inflammation likely contribute to increased CVD risk in PWH and here, we report for the first time, in a randomized placebo-controlled trial of semaglutide, a glucagon-like peptide-1 (GLP-1) receptor agonist, can reduce markers of inflammation in PWH. Levels of hsCRP and IL-6 are related to all-cause mortality in this population [8]; levels of sCD163 are associated with vascular inflammation in PWH [22]. C-reactive protein is an acute-phase reactant that is elevated in metabolic syndrome and T2D, and it has been linked to CVD risk and mortality in PWoH, with levels above 3 µg/mL indicating high risk [23]. Multiple human and animal model studies have demonstrated anti-inflammatory effects of GLP-1 inhibitors, including decreased levels of IL-6, IL-1β, TNFα, and reactive oxygen species during treatment; these effects may be due to modulation of inflammatory signaling pathways, including PPAR-α and nuclear factor-κB, by GLP-1RAs [24]. Studies of semaglutide in PWoH who were obese/overweight [25] or had T2D [26] report similar changes in levels of CRP during semaglutide treatment, and changes in CRP in these studies were related to changes in HbA1c and or body weight. The median value of hsCRP in the semaglutide arm in this study was 4.04 µg/mL, indicating many participants were at elevated CVD risk based on hsCRP levels. Not surprisingly, changes in levels of IL-6 were greater in participants who had baseline levels of hsCRP greater than the median compared to IL-6 changes in individuals with lower levels of hsCRP.

Other studies have sought to reduce chronic inflammation and cardiometabolic risk in PWH with various therapeutic interventions. Results from the REPRIEVE trial show that pitavastatin treatment significantly reduced CVD events in PWH [13] but had modest effects on inflammatory biomarkers, with significant reductions in the vascular inflammation marker lipoprotein-associated phospholipase A2 and oxidized LDL at 24 months, but no significant reduction in IL-6, hsCRP, or sCD163 [12]. We previously reported that rosuvastatin treatment reduced oxidized LDL [27] and lipoprotein-associated phospholipase A2 levels in PWH, as well as decreasing levels of the monocyte activation marker sCD14 and IP-10 [14, 15]. Furthermore, rosuvastatin treatment also reduced expression of the procoagulant molecule tissue factor on “patrolling” CD14^Dim^CD16^+^ monocytes and decreased CD8^+^ and CD4^+^ T-cell activation (CD38 and HLA-DR) [14]. The magnitude of reduction in hsCRP levels reported here (−1.17 µg/mL) is similar to the reduction reported in a trial of IL-6 receptor blockade by tocilizumab (TCZ) in PWH (−1.82 µg/mL) [28]. The TCZ trial also reported decreases in several other plasma markers of immune activation/inflammation (eg, sCD14, D-dimer, TNFRI and II) that have been linked to morbidity and mortality in PWH [8, 29] but TCZ also caused dramatic changes in lipid profiles among participants. Much like our results here, the TCZ trial had minimal effects on T-cell activation profiles. These results demonstrate the complex mechanisms that are likely to contribute to inflammation and cardiometabolic disease in PWH and further study is warranted.

Although the changes in several markers of inflammation following semaglutide treatment are of interest, this study has some limitations that should be considered. First, the trial was of a relatively short duration (32 weeks), which may be insufficient for measurement of robust changes in cellular activation or all circulating biomarkers of inflammation. The semaglutide arm also had lower baseline levels of hsCRP, IL-6, and sICAM-1 compared to levels in the placebo group. Furthermore, although we recognize comparing indices of immune activation across studies may not be ideal, levels of T-cell activation and proportions of CD16^+^ monocyte subset proportions were lower in this study compared to levels we have reported in other studies [14, 30, 31], potentially limiting the likelihood for measurement of significant decreases in these indices. Finally, while only a small proportion of study participants were on statins (3.7% in the semaglutide arm, 7.4% in the placebo arm), future studies should consider the potential additive effects of semaglutide on inflammation and immune activation among PWH receiving statin therapy. Overall, the significant decreases in levels of hsCRP, IL-6, and sCD163 reported here are intriguing and potentially biologically important, but we cannot infer a potential clinical benefit among participants receiving semaglutide. Future studies of GLP-1RAs in PWH should be considered, including those aimed at understanding the mechanisms related to semaglutide's anti-inflammatory effects, whether it improves the concentration and composition of lipid classes or individual lipid species, and assessments of the long-term effects of GLP-1RAs on immune function, cardiovascular risk, and cognitive function in PWH should be considered.

## Supplementary Material

ofaf152_Supplementary_Data

## Supplementary Data

Supplementary materials are available at *Open Forum Infectious Diseases* online. Consisting of data provided by the authors to benefit the reader, the posted materials are not copyedited and are the sole responsibility of the authors, so questions or comments should be addressed to the corresponding author.

## Notes

***Author Contributions.*** All authors reviewed the manuscript and figures. G.M. and A.E. designed the study and obtained funding. Q.W. and A.S. provided statistical support. M.C. and K.A. provided technical support and performed laboratory assays.

***Financial support.*** This work was supported by the National Institutes of Health (NIH; R01DK121619 to G.A.M. and A.R.E.) and from the Clinical and Translational Science Collaborative (CTSC) of Northern Ohio (UM1TR004528 to G.A.M.), which is funded by the National Institutes of Health, National Center for Advancing Translational Sciences. Its contents are solely the responsibility of the authors and do not necessarily represent the official views of the NIH.
