# Supplementary material for: The Effects of Semaglutide on Inflammation and Immune Activation in HIV-associated Lipohypertrophy
Source: Open Forum Infect Dis. 2025 Mar 20;12(4):ofaf152. doi: 10.1093/ofid/ofaf152 (PMC11950536; doi:10.1093/ofid/ofaf152)
Supplement: ofaf152_Supplementary_Data [file ofaf152_supplementary_data.docx]

**Supplemental Tables**

**Supplemental Table 1. Baseline characteristics of immune cell profiles.

Supplemental Table 2. Changes in immune cell subsets and activation over the 32-week study period.** The Absolute changes were computed as (Week 32 – Baseline) and the percentage changes were computed as ((Week 32 – Baseline)/ |Baseline|) x 100.

**Supplemental Table 3A*:* Regression results for the association between changes in inflammatory markers and changes in weight or visceral fat**

**Supplemental Table 3B*:* Regression results for the association between changes in inflammatory markers and changes in weight or visceral fat within Semaglutide group**

|  | **Semaglutide (N=54)** | **Placebo (N=54)** | **P-value** |
| --- | --- | --- | --- |
| cd14p_cd16n_monocyte_population | 0.73 (0.57-0.83) | 0.75 (0.61-0.83) | 0.5909 |
| cd14p_cd16p_monocyte_population | 0.17 (0.11-0.28) | 0.17 (0.12-0.25) | 0.7808 |
| cd14dimcd16p_monocyte_population | 0.08 (0.05-0.13) | 0.08 (0.06-0.12) | 0.6168 |
| cd4p_pd1p | 91.21 (86.72-94.90) | 93.29 (88.28-96.23) | 0.0722 |
| cd8p_pd1p | 64.31 (52.67-72.11) | 68.19 (54.92-78.61) | 0.0763 |
| cd4p_drp_cd38p | 5.92 (3.45-11.54) | 5.80 (4.43-8.39) | 0.8770 |
| cd8p_drp_cd38p | 8.48 (5.47-15.27) | 6.90 (3.96-10.54) | **0.0472** |
| cd4_cd57_pos_cd28_neg | 0.79 (0.00-3.00) | 0.90 (0.03-2.05) | 0.9440 |
| cd8_cd57_pos_cd28_neg | 28.82 (19.70-35.70) | 24.81 (14.27-31.09) | 0.0707 |
| cd4_mfi_pd_pos | 3.78 (3.22-4.39) | 3.82 (3.26-4.56) | 0.6859 |
| cd8_mfi_pd_pos | 2.94 (2.72-3.30) | 2.96 (2.65-3.38) | 0.9320 |
| cd4_mfi_cd38pe | 6.18 (5.56-8.09) | 6.12 (5.12-7.30) | 0.2487 |
| cd8_mfi_cd38pe | 4.84 (3.92-6.49) | 4.46 (3.94-5.64) | 0.6542 |
| cd4_mfi_hladr_fitc | 15.85 (12.64-21.92) | 16.55 (12.73-23.26) | 0.7903 |
| cd8_hladr_mfi | 8.06 (6.31-9.72) | 8.16 (6.30-10.35) | 0.8392 |
| ***Supplemental Table 1:* Baseline characteristics on cellular markers** | | |  |

|  | **Semaglutide (N=54)** | | **Placebo (N=54)** | | **p^**^** |
| --- | --- | --- | --- | --- | --- |
|  | **Median (IQR)** | **p^*^** | **Median (IQR)** | **p^*^** |  |
| 1. **Absolute changes over 32 weeks** | | | | | |
| cd14p_cd16n_monocyte_population | 0.02 (-0.06-0.14) | 0.1017 | 0.01 (-0.08-0.09) | 0.8794 | 0.2050 |
| cd14p_cd16p_monocyte_population | -0.02 (-0.11-0.05) | 0.2026 | 0.00 (-0.10-0.08) | 0.8932 | 0.3021 |
| cd14dimcd16p_monocyte_population | -0.01 (-0.03-0.01) | 0.0578 | -0.01 (-0.04-0.02) | 0.2929 | 0.6305 |
| cd4p_pd1p | 1.70 (-2.84-4.38) | 0.1904 | 0.50 (-1.77-3.62) | 0.3943 | 0.6196 |
| cd8p_pd1p | -0.81 (-7.82-9.77) | 0.8258 | -1.84 (-7.56-4.41) | 0.5286 | 0.6091 |
| cd4p_drp_cd38p | 0.07 (-1.93-3.91) | 0.7605 | 0.92 (-2.44-6.47) | 0.2990 | 0.5994 |
| cd8p_drp_cd38p | -0.63 (-4.95-1.17) | 0.0617 | 0.34 (-1.98-3.23) | 0.4693 | 0.0608 |
| cd4_cd57_pos_cd28_neg | 0.00 (-0.18-1.44) | 0.0868 | 0.34 (-0.01-1.62) | **0.0037** | 0.4358 |
| cd8_cd57_pos_cd28_neg | 2.26 (-9.84-7.23) | 0.6624 | 4.07 (-4.39-11.15) | **0.0334** | 0.2937 |
| cd4_mfi_pd_pos | 0.34 (-0.04-1.50) | **0.0003** | 0.44 (-0.05-1.19) | **0.0015** | 0.6906 |
| cd8_mfi_pd_pos | 0.29 (-0.12-0.82) | **0.0351** | 0.13 (-0.17-0.83) | **0.0244** | 0.8407 |
| cd4_mfi_cd38pe | 0.16 (-1.13-2.11) | 0.4735 | 0.44 (-1.08-2.41) | 0.2339 | 0.6108 |
| cd8_mfi_cd38pe | 0.36 (-0.82-1.55) | 0.3311 | 0.34 (-1.12-1.63) | 0.3823 | 0.9812 |
| cd4_mfi_hladr_fitc | -0.67 (-6.18-6.80) | 0.8307 | -2.30 (-5.36-5.22) | 0.3537 | 0.4166 |
| cd8_hladr_mfi | 0.37 (-0.82-3.96) | 0.0860 | 0.75 (-1.61-3.20) | 0.2763 | 0.8243 |
| 1. **% Changes over 32 weeks** | | | | | |
| cd14p_cd16n_monocyte_population | 3.12 (-5.98-20.00) | 0.0921 | 1.19 (-10.58-14.07) | 0.7350 | 0.2964 |
| cd14p_cd16p_monocyte_population | -11.11 (-46.51-42.86) | 0.9711 | 0.00 (-35.83-64.33) | 0.2073 | 0.3185 |
| cd14dimcd16p_monocyte_population | -14.29 (-37.50-25.00) | 0.2068 | -10.42 (-33.33-58.33) | 0.9534 | 0.4728 |
| cd4p_pd1p | 1.79 (-3.07-4.86) | 0.2082 | 0.52 (-1.83-3.99) | 0.4008 | 0.7058 |
| cd8p_pd1p | -1.12 (-11.94-14.87) | 0.5236 | -2.16 (-11.28-9.29) | 0.5995 | 0.7365 |
| cd4p_drp_cd38p | 2.26 (-35.53-77.73) | 0.2250 | 21.00 (-41.41-119.85) | **0.0357** | 0.5938 |
| cd8p_drp_cd38p | -7.42 (-35.99-34.88) | 0.5997 | 4.29 (-30.90-48.31) | 0.2118 | 0.1892 |
| cd4_cd57_pos_cd28_neg | 15.28 (-60.48-76.65) | 0.3636 | 62.50 (-43.75-143.88) | **0.0053** | 0.3342 |
| cd8_cd57_pos_cd28_neg | 7.76 (-34.40-39.73) | 0.0639 | 19.39 (-14.33-57.19) | **0.0029** | 0.2787 |
| cd4_mfi_pd_pos | 13.00 (-1.95-41.90) | **0.0001** | 10.85 (-1.21-34.13) | **0.0014** | 0.6312 |
| cd8_mfi_pd_pos | 11.60 (-4.96-24.70) | **0.0080** | 6.41 (-6.55-27.26) | **0.0134** | 0.8637 |
| cd4_mfi_cd38pe | 3.02 (-19.02-33.91) | 0.1923 | 7.43 (-15.94-45.18) | 0.0619 | 0.6458 |
| cd8_mfi_cd38pe | 6.82 (-18.93-31.04) | 0.1597 | 8.08 (-20.82-38.49) | 0.1158 | 0.8576 |
| cd4_mfi_hladr_fitc | -3.97 (-35.66-39.18) | 0.5129 | -13.26 (-32.76-51.31) | 0.9037 | 0.5709 |
| cd8_hladr_mfi | 6.86 (-9.37-53.48) | **0.0439** | 10.33 (-17.34-43.08) | 0.0903 | 0.8761 |
| ***Supplemental Table 2:*** **Absolute and percent changes in the cellular markers over the 32-week study period.** | | | | | |

| Inflammatory markers | Weight as a covariate | | VAT as a covariate | |
| --- | --- | --- | --- | --- |
|  | β (SE) | p-value | β (SE) | p-value |
| hsCRP | 0.02 (0.03) | 0.49 | -0.002 (0.01) | 0.84 |
| D-dimer | -0.001 (0.003) | 0.85 | 0.0003 (0.001) | 0.77 |
| sTNFR-I | -0.001 (0.003) | 0.70 | -0.0003 (0.001) | 0.75 |
| sTNFR-II | 0.01 (0.01) | 0.41 | 0.002 (0.003) | 0.44 |
| oxLDL | -0.23 (0.34) | 0.50 | -0.09 (0.12) | 0.46 |
| IL-6 | 0.0004 (0.02) | 0.98 | -0.02 (0.01) | 0.01† |
| sVCAM-1 | -3.15 (1.49) | 0.04 | -0.03 (0.60) | 0.96 |
| sICAM-1 | 0.19 (0.73) | 0.80 | -0.22 (0.21) | 0.30 |
| sCD14 | -7.65 (5.59) | 0.18 | -3.62 (2.17) | 0.10 |
| sCD163 | -0.15 (1.75) | 0.93 | -0.51 (0.62) | 0.42 |

†Significant p-values after Benjamini-Hochberg false discovery rate 0.20 correction.

***Supplemental Table 3A:* Regression results for the association between changes in inflammatory markers and changes in weight or visceral fat**

| Inflammatory markers | Weight as a covariate | | VAT as a covariate | |
| --- | --- | --- | --- | --- |
|  | β (SE) | p-value† | β (SE) | p-value† |
| hsCRP | -0.05 (0.06) | 0.39 | 0.02 (0.03) | 0.45 |
| D-dimer | 0.003 (0.01) | 0.61 | -0.001 (0.002) | 0.74 |
| sTNFR-I | -0.005 (0.004) | 0.21 | -0.001 (0.002) | 0.62 |
| sTNFR-II | 0.005 (0.01) | 0.61 | 0.01 (0.005) | 0.03 |
| oxLDL | -0.29 (0.43) | 0.51 | 0.06 (0.21) | 0.79 |
| IL-6 | -0.01 (0.02) | 0.77 | -0.02 (0.01) | 0.21 |
| sVCAM-1 | -3.21 (1.70) | 0.07 | 0.08 (0.87) | 0.93 |
| sICAM-1 | -0.09 (0.60) | 0.88 | 0.12 (0.30) | 0.68 |
| sCD14 | -10.34 (5.82) | 0.08 | -1.42 (3.57) | 0.69 |
| sCD163 | -0.42 (2.34) | 0.86 | -1.12 (1.16) | 0.34 |

†No significant p-values after Benjamini-Hochberg false discovery rate 0.20 correction.

***Supplemental Table 3B:* Regression results for the association between changes in inflammatory markers and changes in weight or visceral fat within Semaglutide group**
